# Semiquantitative PET Parameters Refine Prognosis in CAR T–Treated Lymphoma After 1 and 3 Months: A Prospective Single-Center Study

**DOI:** 10.2967/jnumed.125.269670

**Published:** 2025-08

**Authors:** Andrea Farolfi, Beatrice Casadei, Claudio Malizia, Riccardo Ussia, Veronica Rocchi, Andrea Paccagnella, Marianna Gentilini, Cristina Nanni, Lisa Argnani, Pier Luigi Zinzani, Stefano Fanti

**Affiliations:** 1 Nuclear Medicine, IRCCS Azienda Ospedaliero-Universitaria di Bologna, Bologna, Italy;; 2 IRCCS Azienda Ospedaliero-Universitaria di Bologna, Istituto di Ematologia “Seragnoli”, Bologna, Italy;; 3 Nuclear Medicine Unit, “M. Bufalini” Hospital, AUSL Romagna, Cesena, Italy; and; 4 Dipartimento di Scienze Mediche e Chirurgiche, Università di Bologna, Bologna, Italy

**Keywords:** hematology, lymphoma, CAR T, prognostic biomarker

## Abstract

Chimeric antigen receptor T-cell (CAR T) therapy has shown remarkable efficacy in treating relapsed or refractory large B-cell lymphoma. However, for nearly half of these patients, the therapy eventually does not achieve durable remission. We investigated whether semiquantitative PET parameters (namely, SUV_max_, metabolic tumor volume [MTV], and total lesion glycolysis [TLG]) could improve risk stratification 1 mo (PET1m) and 3 mo (PET3m) after CAR T infusion. **Methods:** In this prospective, single-center cohort study, patients with large B-cell lymphoma received axicabtagene ciloleucel or tisagenlecleucel. [^18^F]FDG PET/CT scans were acquired at baseline, 1 mo, and 3 mo after infusion. MTV and TLG were calculated using a threshold SUV_max_ of 4 or greater. Patients were followed for overall survival (OS), progression-free survival (PFS), and duration of response (DoR). The imaging assessment was based on the Lugano recommendation for response assessment. Prognostic factors were identified using univariate and multivariate Cox regression. **Results:** Sixty-one patients were enrolled, with a median follow-up of 18 mo. Twenty-eight (46%) patients died. Kaplan–Meier analysis with log-rank tests indicated a significant association of elevated Deauville score (DS), SUV_max_, MTV, and TLG with OS (all *P* < 0.05). DS cutoff was arbitrarily fixed at 4. The optimal SUV_max_, MTV, and TLG cutoffs at PET1m were 9.1, 60.8, and 97.0, respectively; whereas at PET3m, they were 6.3, 120.1, and 436.9, respectively. Patients with an SUV_max_ of 6.3 or greater at PET3m had an 8-fold increase in risk of death (hazard ratio [HR], 8.15; 95% CI, 2.81–23.6; *P* < 0.01) compared with those below this cutoff. Similarly, higher MTV (≥120.1) at PET3m yielded a nearly 10-fold risk (HR, 9.87; 95% CI, 3.65–26.7; *P* < 0.01). DS, SUV_max_, MTV, and TLG at both PET1m and PET3m were associated with OS and PFS (all *P* < 0.05), whereas PET3m parameters also correlated with DoR (*P* < 0.05). Harrell C-index values were higher for PET3m measures than for PET1m, though differences were not statistically significant (*P* > 0.05). On multivariable analysis, older age (HR, 1.10), bridging therapy (HR, 10.91), elevated lactate dehydrogenase (HR, 6.43), increased fibrinogen (HR, 5.27), and higher SUV_max_ at PET3m (HR, 11.03) independently predicted poorer OS. There were no significant associations between SUV_max_, MTV, and TLG with CAR T–related toxicities. **Conclusion:** Semiquantitative PET parameters, such as SUV_max_, MTV, and TLG, at 1 mo and 3 mo after CAR T–cell therapy correlate significantly with OS, PFS, and DoR. [^18^F]FDG PET/CT at 3 mo may offer slightly stronger prognostic discrimination, but both time points can be used for early risk stratification.

## Visual Abstract

**Figure:**
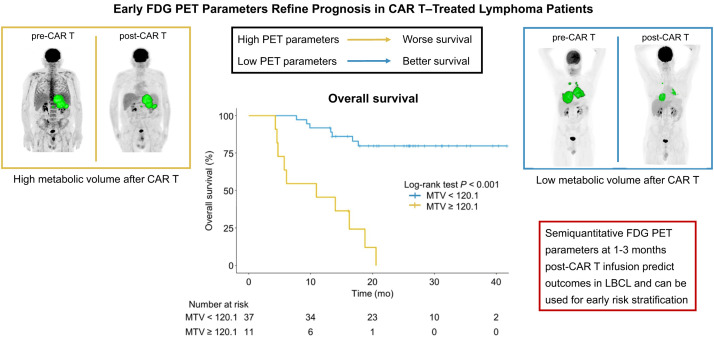

Chimeric antigen receptor T-cell (CAR T) therapy has emerged as a groundbreaking treatment modality for patients with relapsed or refractory large B-cell lymphoma (LBCL) (1–3). This innovative approach harnesses the power of genetically engineered T lymphocytes to specifically target and eliminate malignant cells regardless of major histocompatibility complex, offering a new hope to patients with an otherwise poor prognosis. Despite its remarkable efficacy, with many patients achieving complete remission (CR), the response to CAR T therapy remains heterogeneous (4). Unfortunately, nearly half of the patients experience disease progression or relapse within the first year after treatment, underscoring the need for reliable prognostic tools to identify those at risk of poor outcomes. [^18^F]FDG PET/CT is a well-established imaging technique commonly used to assess metabolic activity in malignancies, including LBCLs. In the context of CAR T therapy, [^18^F]FDG PET/CT is frequently used to evaluate treatment response, with the Deauville score (DS), a visual 5-point scale, being the standard tool for this purpose. However, whereas the DS provides valuable insights into the overall metabolic activity after treatment, its utility in predicting long-term outcomes such as overall survival (OS) and progression-free survival (PFS) has not yet been determined in patients with lymphoma who are treated with CAR T therapy (5*,*^6^). To enhance the predictive accuracy of [^18^F]FDG PET/CT in this setting, there is growing interest in the application of semiquantitative PET parameters. Metrics such as SUV_max_, metabolic tumor volume (MTV), and total lesion glycolysis (TLG) offer a more detailed quantification of tumor metabolism and burden, which could potentially refine the assessment of treatment response and provide prognostic information. However, CAR T therapy is also associated with toxicity, including cytokine release syndrome (CRS) and immune effector cell–associated neurotoxicity syndrome (ICANS), which can be life-threatening (7–9). Prior studies have explored the relationship between PET parameters and toxicity outcomes with mixed results. For instance, Wang et al. reported an association between higher MTV on baseline PET and severe CRS (10), whereas Iacoboni et al. found no link between baseline MTV and CRS but noted an association between higher MTV and shorter PFS (11). Conversely, Cohen et al. did not identify any correlations between baseline PET parameters and CRS or ICANS (5). To the best of our knowledge, there is a lack of studies investigating toxicity and postinfusion PET parameters at either 1-mo or 3-mo intervals. By analyzing SUV_max_, TLG, MTV, and DS values obtained at 1 mo (PET1m) and that taken at 3 mo (PET3m) after CAR T infusion, and correlating these metrics with the OS, PFS, and duration of response (DoR), this research seeks to determine the potential of advanced PET/CT metrics in guiding post-CAR T–therapy management. The findings could provide crucial insights for clinicians, enabling more personalized and timely interventions to improve patient prognosis in this challenging cohort. Therefore, this study aims to investigate the prognostic value of DS and semiquantitative PET parameters (SUV_max_, TLG, and MTV) measured at 1- and 3-mo after CAR T in predicting long-term outcomes for LBCL patients. Our secondary aim is to investigate which time point for early PET assessment is more informative, that is, PET1m or PET3m. An exploratory aim is to investigate the utility of PET parameters (i.e., SUV_max_, MTV, and TLG) in identifying patients at risk for CRS and ICANS.

## MATERIALS AND METHODS

### Study Design and Population

The study population was based on a prospective cohort of 61 consecutive patients who were treated at our institution with commercial CAR T products (axicabtagene ciloleucel [axi-cel] or tisagenlecleucel [tisa-cel]) between August 2019 and September 2022. The following inclusion criteria were applied: patients no older than 75 y with diffuse large B-cell lymphoma (DLBCL), high-grade B-cell lymphoma or primary mediastinal LBCL (PMBCL) relapsed after or refractory to at least 2 previous lines of therapy; patients having [^18^F]FDG PET/CT imaging studies at baseline (bPET, that is, within 7 d of lymphodepletion), at PET1m and PET3m after infusion; and patients being available for follow-up. All patients underwent leukapheresis, followed by CAR T infusion. Bridging therapy (BT), given at the discretion of the treating physician to control or debulk the disease, was defined as systemic therapy done between the time of leukapheresis and the time of CAR T infusion. The decision to use axi-cel or tisa-cel was dependent only on slot production availability and histology, based on each product approval. Patients received lymphodepletion for 3 d before CAR T infusion, with intravenous fludarabine and cyclophosphamide according to the manufacturers’ instructions. The main CAR T–associated toxicities, that is, CRS and ICANS graded according to the American Society for Transplantation and Cellular Therapy criteria (9), were correlated with semiquantitative PET parameters. Follow-up visits after CAR T therapy were scheduled at 1, 3, 6, 12, 18, and 24 mo after infusion.

This study was approved by our institutional board (Ethical Committee AVEC of Bologna, approval 503/2024/Oss/AOUBo). All participants gave written informed consent (when applicable) in accordance with the Declaration of Helsinki to collect their data.

### PET/CT Images

All patients fasted for at least 6 h before [^18^F]FDG administration, had blood glucose levels measured and maintained below 150 mg/dL (per European Association of Nuclear Medicine [EANM] guidelines), and then remained at rest for approximately 60 min between tracer injection and image acquisition (12). PET/CT images were acquired 60 min after tracer injection (159–275 MBq weight-adapted with ∼2.5–4.5 MBq of [^18^F]FDG per kg of body weight) and for the [^18^F]FDG PET/CT–unenhanced CTs using a slice thickness of 2 mm, 120 kVp, 100–400 mAs, and dose modulations were performed for attenuation correction. We used Discovery MI (GE HealthCare) and uMI Vista (United Imaging Health Care) scanners. Both scanners fulfilled the requirements indicated in the EANM imaging guidelines and obtained EANM Research Ltd. accreditation during acquisition. The following reconstruction algorithms were used: for GE Discover MI scanner, 8 subsets, 4 iterations; for uMI Vista scanner, 20 subsets, 2 iterations. All systems resulted in a PET image with a voxel size of 2 × 2 × 2 mm^3^. Images were normalized to decay-corrected injected activity per kilogram of body weight.

### Imaging Assessment

The MTV and TLG were determined as follows: attenuation-corrected PET images were analyzed using MIM software with an absolute threshold of SUV (SUV_max_ ≥ 4) to define hypermetabolic lymphoma tissue as described before; these studies have also demonstrated the reproducibility of this approach (13–15). A reader with 4 y of experience in hematology and nuclear medicine performed the initial manual correction, which was subsequently reviewed by 2 senior physicians with 8 y of experience in nuclear medicine and lymphoma. SUV_max_, MTV, TLG, number, and anatomic location of all lymphomatous lesions were assessed for each PET/CT, that is, bPET, PET1m, and PET3m. Lesions were categorized as either nodal, spleen, bone, parenchyma (e.g., liver, lung), or soft tissue (e.g., subcutaneous, muscle), and MTV and TLG were calculated. The mesenteric disease was defined as nodal. Discrete, avid bone lesions were contoured and included, but diffuse uptake indistinguishable from marrow was not included. SUV_max_, MTV, and TLG differences between bPET and either PET1m or PET3m were calculated. The imaging assessment was based on the Lugano recommendation for response assessment (16). Response was assessed locally according to the 5-point DS system. If the PET scan suggested disease progression, a biopsy confirmation was performed when clinically indicated. Transient response was defined as progressive disease (PD) by month 3 or month 6 after CR or partial response (PR) at the 1-mo assessment.

### Statistical Analysis

Baseline patient characteristics and PET/CT parameters were summarized as counts and percentages, median and interquartile range (IQR), or mean and SD. When appropriate, continuous PET values were dichotomized to create scientifically appropriate groups. Lugano classification metabolic response categories were applied for CR, PR, stable disease, and PD. Also, patients were dichotomized in PD and non-PD, that is, patients with stable disease and PD versus CR and PR. The primary endpoint of the study was the optimal cutoff of PET-derived parameters, that is, DS, SUV_max_, MTV, and TLG, at 1 and 3 mo after infusion for OS, PFS, and DoR. OS was calculated from the date of infusion until death as a result of any cause or last follow-up. PFS was defined as the time from infusion for all treated patients to the first observation of PD or death as a result of any cause (17). DoR is from the time when criteria for response (i.e., CR or PR) are met, for which the event is the first documentation of relapse or progression. For each endpoint (OS, PFS, and DoR), the best cutoff values (Youden index) for the semiquantitative PET variables, including SUV_max_, MTV, and TLG, were identified through the receiver operating characteristic curve analysis at both 1 mo and 3 mo. Cutoff for DS was predefined as 4 (DS < 4 and DS ≥ 4). These cutoff values were calculated to dichotomize the semiquantitative variables and the DS into 2 groups for Kaplan–Meier analyses and Cox regressions. Kaplan–Meier survival curves were generated to estimate OS, PFS, and DoR. The log-rank test was used to compare the endpoints of OS, PFS, and DoR distributions between dichotomized groups. Univariate Cox proportional hazard regression analysis was performed to evaluate the association between each semiquantitative PET variables, both continuous and dichotomized, and the endpoints OS, PFS, and DoR. Variables with a *P* value of less than 0.2 in the univariate analysis were included in the multivariate Cox regression model to identify independent prognostic factors. The Harrell C-index was calculated to evaluate the predictive accuracy of semiquantitative PET parameters for each endpoint of OS, PFS, and DoR. The C-index was calculated as the proportion of concordant pairs among all evaluable pairs, adjusted for tied pairs. Laboratory parameters and CAR-HEMATOTOX clinical score (18) were analyzed, and Kaplan–Meier survival curves were constructed to evaluate outcomes. Continuous variables such as SUV_max_, MTV, and TLG were compared between patients with PD and non-PD using the nonparametric Mann–Whitney test in view of the asymmetric distribution of the variables. SUV_max_, MTV, and TLG were analyzed against the CRS and ICANS variables using the Wilcoxon statistical test. The analyses were performed with the statistical software R (version 4.3.2; https://www.r-project.org/) using a *P* value of less than 0.05 as a threshold of statistical significance.

## RESULTS

### Patient Characteristics

**FIGURE 1.**
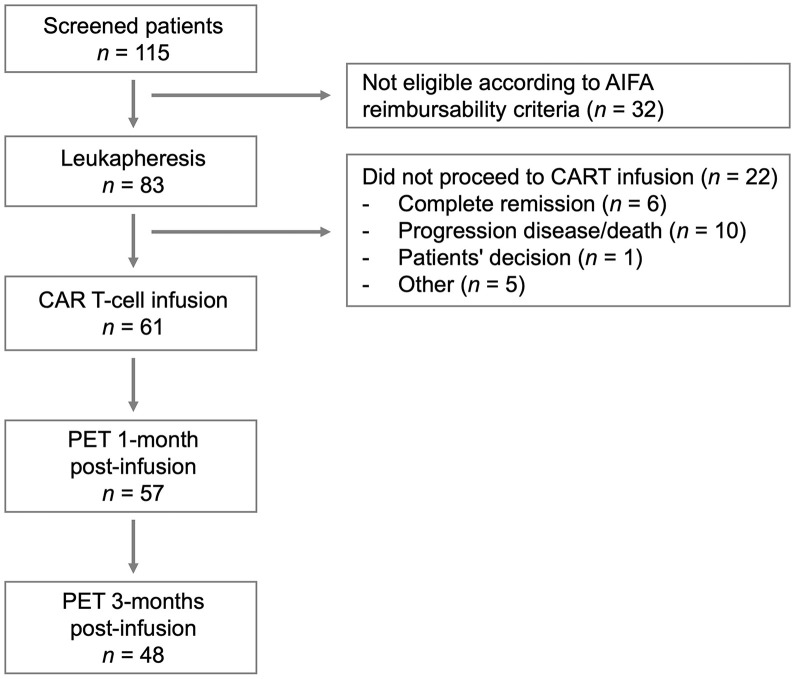
Consort diagram for patient selection.

Sixty-one patients matched the inclusion criteria (median age, 59 y; 30% women). A flow chart is provided in Figure 1. Fifty-three (87%) patients presented with DLBCL and 8 (13%) patients with PMBCL (Table 1). Forty-six of 61 (75%) patients had BT. Twenty-seven (44%) patients received tisa-cel, and 34 (56%) received axi-cel. Most patients had advanced (stage III/IV in 67%) and bulky disease (51%) before apheresis. On bPET, 16 (26%) patients had bone involvement whereas 21 (34%) had visceral disease. All patients were pretreated, with a median number of previous lines of therapy of 2 (IQR, 2–3). At a median follow-up of 18 mo (IQR, 9–26 mo), 26 (43%) patients died. [^18^F]FDG PET/CT scans were performed at 1 mo (PET1m: median, 31 d; IQR, 30–34 d) and 3 mo (PET3m: median, 91 d; IQR, 86–95 d) after infusion. The mean bPET MTV was 477.5 mL and TLG was 3,205.5 g (Table 2). After 1 mo, the mean PET1m MTV was 153.5 mL and TLG was 1,475.0 g, whereas after 3 mo, the mean PET3m MTV was 186.7 mL and TLG was 1,301.1 g. The description of the functional PET/CT parameters studied, SUV_max_, MTV, and TLG, is depicted in Table 3, which summarizes the results of the receiver operating characteristic analysis used to identify optimal cutoff points. Based on Lugano classification metabolic response categories, the overall response rate at PET1m was 67% with 38% obtaining a CR. At PET3m, the overall response rate was 77% with 58% having a CR compared with bPET (Supplemental Fig. 1; supplemental materials are available at http://jnm.snmjournals.org). Regarding transient responders, 6 of 39 (15%) responder patients at 1 mo showed PD at 3 mo, and 14 of 39 (36%) showed PD at 6 mo. On the other hand, nonresponder patients at 1 mo were 22 of 61 (36%), of whom 4 (18%) showed CR or PR at 3 mo and 1 (5%) showed CR or PR at 6 mo. Overall, of the 61 patients, 31 (51%) exhibited PD on [^18^F]FDG PET/CT. Among these, 29 of 31 (94%) underwent biopsy to confirm disease progression, all of which were positive for lymphoma, yielding a 100% true positive rate. There was statistically significant difference between DS, SUV_max_, MTV, and TLG of PD versus the non-PD group at PET1m (all *P* < 0.01) and at PET3m (all *P* < 0.01) (Supplemental Fig. 2).

**TABLE 1.** Demographic and Clinical Characteristics of Patients

Characteristic	Patients (*n* = 61)
Age (y)	59 (48–65)
Sex	
Female	18 (30)
Male	43 (70)
Disease stage at study entry	
I	1 (2)
II	19 (31)
III	8 (13)
IV	33 (54)
Diagnosis on central histologic review	
DLBCL	53 (87)
PMBCL	8 (13)
High-grade B-cell lymphoma	0
Double- or triple-hit rearrangement: MYC plus BCL2, BCL6, or both (*n* = 53)	6 (11)
Cell of origin of cancer	
Germinal center B-cell type	16 (26)
Nongerminal center B-cell type	28 (46)
Activated B-cell	2 (3)
Missing data	15 (25)
Number of previous lines of antineoplastic therapy	
1	1 (2)
2	33 (54)
3	16 (26)
4–8	11 (18)
Relapse after last therapy	6 (10)
Refractory DLBCL	55 (90)
CAR-T product	
Axi-cel	34 (56)
Tisa-cel	27 (44)

Data are number with percentage in parentheses. Continuous data are median with IQR in parentheses.

**TABLE 2.** PET Parameters for bPET, PET1m, and PET3m

	SUV_max_	MTV	TLG
bPET	18.3 (9.7–25.2)	477.5 (30.3–399.9)	3,205.5 (185.1–2,837.0)
PET1m	8.2 (0–13.6)	153.5 (0–54.6)	1,475.0 (0–391.0)
PET3m	5.9 (0–10.2)	186.7 (0–12.1)	1,301.1 (0–59.3)

Continuous data are median and IQR in parentheses.

**TABLE 3.** Metabolic PET/CT Parameter Cutoff Calculated Using Receiver Operating Characteristic (ROC) Curve Analysis Considering the Entire Cohort

	ROC curve for OS					ROC curve for PFS					ROC curve for DoR				
Parameter	Cutoff	AUC (95% CI)	*P*	Sensitivity	Specificity	Cutoff	AUC (95% CI)	*P*	Sensitivity	Specificity	Cutoff	AUC (95% CI)	*P*	Sensitivity	Specificity
PET1m															
DS	4					4					4				
SUV_max_	9.1	0.736	0.0007	54%	88%	8.9	0.719	<0.0001	47%	91%	3.3	0.581	0.075	0.562	0.652
MTV	60.8	0.744	<0.0001	50%	94%	48.3	0.748	<0.0001	44%	100%	0.3	0.581	0.16	0.5	0.696
TLG	97.0	0.731	0.00065	58%	85%	204.6	0.731	<0.0001	47%	96%	0.5	0.571	0.18	0.562	0.609
PET3m															
DS	4					4					4				
SUV_max_	6.3	0.799	<0.0001	71%	87%	11.4	0.734	<0.0001	48%	100%	6.3	0.696	<0.0001	0.467	0.913
MTV	120.1	0.805	<0.0001	59%	97%	120.1	0.722	<0.0001	44%	100%	120.1	0.681	<0.0001	0.333	1
TLG	436.9	0.803	<0.0001	59%	97%	67.8	0.725	<0.0001	48%	100%	53.5	0.684	<0.0001	0.4	0.956

Cutoff of 4 for DS is clinical choice.

### Overall Survival

**FIGURE 2.**
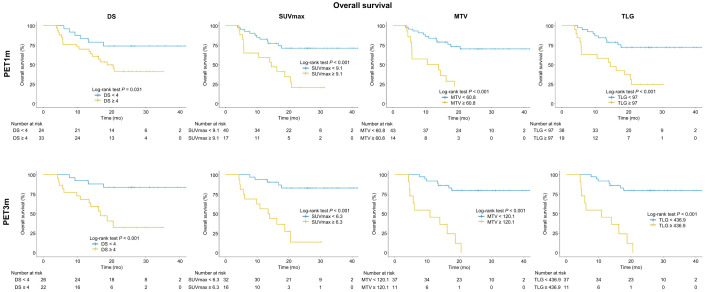
Kaplan–Meier curves for OS, comparing groups dichotomized by DS, SUV_max_, MTV, and TLG at 1 mo and 3 mo.

The median OS was 17 mo (IQR, 9–26 mo), and 28 (45%) patients died during follow-up with an OS of 67% at 2 y. Regarding PET1m and PET3m parameters, a higher DS, SUV_max_, MTV, and TLG were associated with a worse OS (all *P* < 0.05). Log-rank test on Kaplan–Meier analysis showed a statistically significant association of high DS, SUV_max_, MTV, and TLG with OS (all *P* < 0.05) (Table 3; Fig. 2). In univariate analysis of the PET1m and PET3m parameters as continuous variables (Cox regression), a significantly higher risk of death was associated with an increase in SUV_max_, MTV, and TLG for both PET1m and PET3m (Table 4). On univariate analysis of dichotomized PET1m parameters, high DS (hazard ratio [HR], 2.66), SUV_max_ (HR, 3.26), MTV (HR, 3.18), and TLG (HR, 2.82) were each significantly associated with worse OS (all *P* < 0.05) (Table 5). Similarly, for PET3m, elevated DS (HR, 5.53), SUV_max_ (HR, 8.15), MTV (HR, 9.87), and TLG (HR, 7.44) were associated with shorter OS (all *P* < 0.05).

**TABLE 4.** Cox Regression (Continuous PET Parameters)

	OS				PFS				DoR			
Parameter	Beta	HR (95% CI)	Wald test	*P*	Beta	HR (95% CI)	Wald test	*P*	Beta	HR (95% CI)	Wald test	*P*
PET1m												
SUV_max_	0.062	1.0644 (1.03–1.1)	12.2	<0.001	0.11	1.1175 (1.07–1.16)	30.3	<0.001	0.046	1.0466 (0.947–1.16)	0.8	0.372
MTV	0.0016	1.0016 (1–1)	15.8	<0.001	0.0019	1.0019 (1–1)	25.6	<0.001	0.0051	1.0051 (0.997–1.01)	1.7	0.193
TLG	0.00012	1.00012 (1–1)	11.3	<0.001	0.00014	1.0001 (1–1)	16.5	<0.001	−0.00015	0.9998 (0.999–1)	0.22	0.641
PET3m												
SUV_max_	0.081	1.0839 (1.04–1.13)	13.6	<0.001	0.12	1.1268 (1.08–1.18)	26.5	<0.001	0.16	1.1697 (1.09–1.26)	18.7	<0.001
MTV	0.0012	1.00119 (1–1)	9.1	<0.001	0.00089	1.00099 (1–1)	12.6	<0.001	0.0098	1.0099 (1–1.02)	12.8	<0.001
TLG	0.0002	1.0002 (1–1)	12.0	<0.001	0.00015	1.00015 (1–1)	14.3	<0.001	0.0022	1.0022 (1–1)	16.9	<0.001

**TABLE 5.** Cox Regression with Cutoff (Dichotomized PET Parameters)

	OS				PFS				DoR			
Parameter	Beta	HR (95% CI)	Wald test	*P*	Beta	HR (95% CI)	Wald test	*P*	Beta	HR (95% CI)	Wald test	*P*
PET1m												
SUV_max_	1.2	3.26 (1.22–8.75)	5.51	<0.05	1.2	3.23 (1.5–6.98)	8.92	<0.01	0.88	2.42 (0.89–6.55)	3.0	0.08
MTV	1.2	3.18 (1.26–8.03)	5.98	<0.05	1.2	3.44 (1.63–7.24)	10.5	<0.01	0.7	2.01 (0.75–5.39)	1.92	0.16
TLG	1.0	2.82 (1.05–7.56)	4.23	<0.05	1.2	3.33 (1.5–7.41)	8.74	<0.01	0.67	1.95 (0.72–5.3)	1.72	0.19
DS	0.98	2.66 (1.06–6.72)	4.3	<0.05	1.1	3.0 (1.42–6.32)	8.35	<0.01	0.67	1.95 (0.73–5.24)	1.76	0.18
PET3m												
SUV_max_	2.1	8.15 (2.81–23.6)	15	<0.01	2.2	8.63 (3.68–20.2)	24.5	<0.01	2.0	7.15 (2.45–20.9)	12.9	<0.01
MTV	2.3	9.87 (3.65–26.7)	20.4	<0.01	3.5	33.7 (8.68–130.0)	25.9	<0.01	3.7	39.6 (7.29–215.0)	18.2	<0.01
TLG	2.0	7.44 (2.71–20.4)	15.2	<0.01	2.6	13.1 (5.4–31.8)	32.4	<0.01	2.4	11.0 (3.59–33.6)	17.6	<0.01
DS	1.7	5.53 (1.78–17.2)	8.78	<0.01	1.4	4.19 (1.82–9.64)	11.4	<0.01	1.3	3.67 (1.32–10.2)	6.2	<0.05

### PFS

**FIGURE 3.**
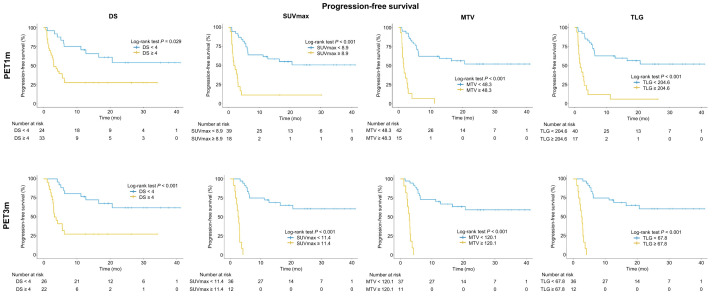
Kaplan–Meier curves for PFS, comparing groups dichotomized by DS, SUV_max_, MTV, and TLG at 1 mo and 3 mo.

The median PFS was 6 mo (IQR, 2–19 mo). Regarding PET1m and PET3m parameters, a higher DS, SUV_max_, MTV, and TLG were associated with a worse PFS (all *P* < 0.05). The log-rank test on Kaplan–Meier analysis showed a statistically significant association of dichotomized DS, SUV_max_, MTV, and TLG with PFS (all *P* < 0.05) (Table 3; Fig. 3). In univariate analysis of the PET1m and PET3m parameters as continuous variables (Cox regression), a significantly higher risk of progression was associated with an increase in SUV_max_, MTV, and TLG for either PET1m or PET3m (Table 4). Univariate analysis of dichotomized parameters for PET1m showed a statistically significant association of high DS (HR, 3.0), SUV_max_ (HR, 3.23), MTV (HR, 3.44), and TLG (HR, 3.33) with worse PFS (all *P* < 0.001) (Table 5). Similarly, for PET3m, elevated DS (HR, 4.19), SUV_max_ (HR, 8.63), MTV (HR, 33.7), and TLG (HR, 13.1) were associated with shorter PFS (all *P* < 0.05).

### DoR

**FIGURE 4.**
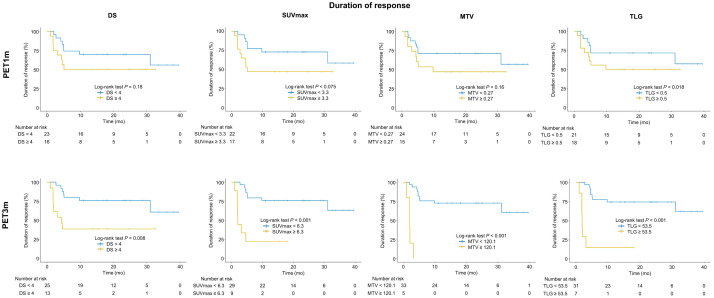
Kaplan–Meier curves for DoR, comparing groups dichotomized by DS, SUV_max_, MTV, and TLG at 1 mo and 3 mo.

Regarding PET1m and PET3m parameters, a higher SUV_max_, MTV, and TLG were associated with a short DoR (all *P* < 0.05), whereas DS was not. The log-rank test on Kaplan–Meier analysis showed a statistically significant association of dichotomized DS, SUV_max_, MTV, and TLG with PFS for PET3m (all *P* < 0.05) but not for PET1m parameters (Table 3; Fig. 4). In univariate analysis of the parameters as continuous variables (Cox regression), a significantly shorter duration of response was associated with an increase in PET3m SUV_max_, MTV, and TLG (all *P* < 0.01) but not for PET1m parameters (Table 4). When PET-derived parameters were simply dichotomized as high or low, patients with higher values of DS (HR, 3.67), SUV_max_ (HR, 7.15), MTV (HR, 39.6), and TLG (HR, 11.0) experienced a shorter duration of response for PET3m (all *P* < 0.05). This association was not seen for PET1m parameters (Table 5).

### Clinical and Laboratory Characteristics

Forty-six (75%) patients received BT. Patients receiving BT had worse OS (*P* = 0.034) but not shorter PFS or DoR. With respect to laboratory parameters, elevated preapheresis levels of lactate dehydrogenase (LDH) and fibrinogen were significantly associated with poorer OS (all *P* < 0.05) but showed no correlation with PFS or DoR. Additionally, a high CAR-HEMATOTOX score was significantly associated with shorter PFS (*P* < 0.05) but not with OS or DoR (Supplemental Fig. 3). Among the 61 patients infused, 51 (84%) developed a CRS of any grade. In most cases, CRS was grade 1–2, with 5 of 61 patients (8%) experiencing CRS grade 3–4. Twenty-one patients (34%) developed ICANS (any grade): 10 (48%) and 11 (52%) had grade 1–2 and 3 or more ICANS, respectively. CRS and ICANS were not associated with SUV_max_, MTV, and TLG at PET1m and PET3m (all *P* > 0.05).

### Subgroup Analyses

Additional analyses to compare outcomes by LBCL subtype (PMBCL vs. DLBCL) as well as by CAR T product (axi-cel vs. tisa-cel) were performed. Neither the log-rank tests nor the univariate Cox regressions indicated any statistically significant differences in OS, PFS, or DoR across these subgroups (all *P* > 0.05). The HRs comparing PMBCL to DLBCL were 0.3 (*P* = 0.46), 0.56 (*P* = 0.28), and 0.29 (*P* = 0.24) for OS, PFS, and DoR, respectively. The HRs comparing axi-cel to tisa-cel were 1.21 (*P* = 0.61) for OS, 0.91 (*P* = 0.79) for PFS, and 2.35 (*P* = 0.09) for DoR.

### Comparative Prognostic Performance

The models with SUV_max_, MTV, and TLG at PET3m yielded a higher Harrell C-index for OS, PFS, and DoR compared with those at PET1m, although these differences did not reach statistical significance (*P* > 0.05). Specifically for OS, SUV_max_ improved from 0.670 at PET1m to 0.752 at PET3m; MTV from 0.678 at PET1m to 0.755 at PET3m; and TLG from 0.668 at PET1m to 0.780 at PET3m (Supplemental Fig. 4).

### Multivariable Analysis

On multivariable analysis combining clinical, laboratory, and PET data, independent factors associated with poor OS were older age (HR, 1.1; 95% CI, 1.02–1.19; *P* < 0.05), BT (HR, 10.91; 95% CI, 1.08–110.38; *P* < 0.05), increased LDH (HR, 6.43; 95% CI, 1.93–21.41; *P* < 0.01), elevated fibrinogen (HR, 5.27; 95% CI, 1.29–21.51; *P* < 0.05), and higher PET3m SUV_max_ (HR, 11.03; 95% CI, 2.79–43.57; *P* < 0.01). Similarly, PET3m SUV_max_ significantly correlated with PFS (HR, 66.17) and DoR (HR, 14.57) after adjusting for clinical factors. Multivariable analyses for OS, PFS, and DoR are displayed on Table 6.

**TABLE 6.** Multivariable Analysis of Clinical and PET-Derived Data

	OS			PFS			DoR		
Parameter	HR	95% CI	*P*	HR	95% CI	*P*	HR	95% CI	*P*
Age	1.1	1.02–1.19	0.014	—	—	—	—	—	—
BT	10.91	1.08–110.38	0.043	3.52	0.98–12.63	0.05	9.69	1.06–88.17	0.04
LDH	6.43	1.93–21.41	0.002	3.10	1.31–7.35	0.01	—	—	—
Fibrinogen	5.27	1.29–21.51	0.021	—	—	—	—	—	—
Ferritin	—	—	—	—	—	—	0.33	0.11–1.0	0.05
SUV_max_*	11.03	2.79–43.57	<0.001	66.17	13.07–335.04	<0.001	14.57	3.91–54.24	<0.001

* SUV_max_ at PET3m.

## DISCUSSION

Our findings reinforce the growing body of evidence suggesting that semiquantitative PET parameters offer valuable prognostic information beyond the traditional DS in assessing the response to CAR T therapy in patients with relapsed or refractory LBCLs. As CAR T therapy use and indications increase, methods of prognostic differentiation using noninvasive techniques such as [^18^F]FDG PET/CT are needed to identify which patients are at the highest risk of PD, reduction in treatment duration, and death, thereby indicating which patients may benefit from treatment intensification or modification and increased monitoring. Previous studies have established the prognostic significance of baseline [^18^F]FDG PET/CT before CAR T infusion. Dean et al. demonstrated that higher MTV on baseline [^18^F]FDG PET/CT is associated with increased risk of PD and death (19). Similarly, Breen et al. found that an increase in MTV and TLG between preleukapheresis and prelymphodepletion chemotherapy [^18^F]FDG PET/CT scans correlates with worse outcomes (20). Extending these findings, Galtier et al. reinforced the prognostic role of baseline MTV in LBCL but additionally showed that a 1-mo post–CAR T [^18^F]FDG PET further refines risk among both low- and high-volume patients (21). Regarding early [^18^F]FDG PET/CT after CAR T infusion, Kuhnl et al. showed that posttreatment DS categories at 1 mo are associated with durable remission, informing early treatment decisions and response-adapted stratification in clinical trial (22). Similarly, another retrospective study on 53 patients confirmed that findings obtained 1 mo after CAR T therapy show accuracy for early response evaluation and prediction of progression in patients with DLBCL (23). Most recently, a prospective study by Guidetti et al. showed that combining DS with quantitative SUV changes at 1 mo can identify patients at high risk of early relapse who might benefit from prompt salvage interventions (24). Our study further corroborates these findings, demonstrating that semiquantitative parameters such as SUV_max_, MTV, and TLG, measured at both 1 and 3 mo after infusion, are prognostic for OS and PFS and also for DoR in a prospective cohort of patients. For early response assessment after CAR T infusion, there is no consensus regarding the timing for [^18^F]FDG PET/CT, indeed both 1-mo and 3-mo time points after therapy infusion are proposed (25). We assessed the prognostic significance of early [^18^F]FDG PET/CT after CAR T infusion, both at 1 mo and 3 mo after infusion. On multivariable analysis combining clinical and PET-derived data, among all independent predictors for OS, PFS, and DoR, the SUV_max_ at 3 mo was the independent predictor with the highest HR. Although both 1-mo and 3-mo posttreatment [^18^F]FDG PET/CT scans provide valuable prognostic information, our data suggest that 3-mo [^18^F]FDG PET/CT may offer slightly more robust predictive power for OS, PFS, and DoR, although the observed increases in the Harrell C-index did not reach statistical significance. These findings indicate that both time points can be feasible and useful for extrapolating prognostic information in terms of quantitative parameters. However, in this study, the prognostic performance of [^18^F]FDG PET/CT at 1 mo yielded a Harrell C-index of 0.653, indicating moderate discriminative ability. Notably, the C-index for [^18^F]FDG PET/CT at 3 mo improved to 0.740, suggesting a stronger capacity to distinguish between patients with different survival outcomes. This enhancement could be attributed to more robust metabolic or disease-related changes captured after 3 mo, thereby offering better prognostic insight and potentially aiding in risk stratification. It is noteworthy to mention that 15% of responder patients at 1 mo showed PD at 3 mo, and around one third showed PD at 6 mo, meaning that transient responders occur at 1 mo. Also, biopsy in PET findings suspected for PD confirmed the disease in 100% of cases.

Our findings support the incorporation of semiquantitative PET parameters derived 1 and 3 mo after CAR T infusion into routine clinical practice for assessing response to CAR T therapy in patients with LBCL. By providing a more comprehensive and objective assessment of tumor metabolism and burden, these metrics could enable clinicians to identify patients at high risk of treatment failure early on, facilitating timely interventions such as histologic confirmation by lymph node biopsy and subsequent salvage therapy or clinical trials. It seems feasible to perform an early scan at 1 mo post–CAR T infusion to stratify patients at higher risk of progression and for early evaluation of further treatment options. For patients with no evidence of progression or without any other treatment option available after CAR T therapy, [^18^F]FDG PET/CT at 3 mo may be the optimal choice. Additional prognostic factors for OS identified in our analysis included preapheresis laboratory parameters such as LDH and fibrinogen, whereas a high CAR-HEMATOTOX score correlated with shorter PFS but not OS or DoR. Elevated LDH levels have previously been established as an independent predictor of reduced OS in other studies (5*,*^26^). Furthermore, no significant associations were observed between CRS or ICANS and any PET-derived parameters, suggesting that these syndromes may involve subtle mechanisms and are not influenced by PET semiquantitative parameters such as SUV_max_, MTV, and TLG calculated at 1 and 3 mo after CAR T therapy. Additionally, it is notable that our subgroup analyses did not reveal any significant differences in outcomes among LBCL subtypes (PMBCL vs. DLBCL) or between CAR T products (axi-cel vs. tisa-cel), suggesting that, within the limits of this cohort, neither lymphoma subtype nor product choice exerted a major influence on OS, PFS, or DoR.

Although our study adds valuable insights to the field, it is essential to acknowledge certain limitations. First, the relatively small sample size might limit the generalizability of our findings, even if in a prospective fashion. Second, although we included a diverse range of clinical and laboratory characteristics in our analysis, other factors such as patient comorbidities and prior treatment history could also influence outcomes and warrant further investigation. Despite these limitations, our study is strengthened by its single-center design that ensures consistency in PET imaging acquisition protocols, contouring methods, and clinical decision-making, minimizing variability and enhancing the reliability of the findings; the long follow-up time with a median of 18 mo in a setting of disease in which the prognosis is very poor, was estimated to be only 4–6 mo (27*,*^28^). Most importantly, DS was evaluated together with SUV_max_, MTV, and TLG at 2 time points after CAR-T therapy, providing initial evidence of their prognostic value at 1 and 3 mo after infusion. Further research is needed to validate these findings in larger, multicenter cohorts and to explore the potential of PET-guided treatment algorithms to optimize outcomes in this patient population.

Our study underscores the prognostic value of semiquantitative PET parameters in predicting clinical outcomes after CAR T therapy for LBCL. These findings suggest that PET-based metrics should be considered alongside the DS to hasten treatment decisions in high-risk patients and optimize their management. Either [^18^F]FDG PET/CT 1 or 3 mo after CAR T infusion may be used.

## CONCLUSION

Early [^18^F]FDG PET/CT assessment at either 1 mo or 3 mo after CAR T infusion in patients with LBCL provides prognostic information, and [^18^F]FDG PET/CT at 3 mo provides enhanced prognostic discrimination compared with 1 mo. Further prospective studies are required to confirm whether integrating semiquantitative PET parameters into routine practice ultimately informs treatment decisions and improves patient outcomes.

## DISCLOSURE

The work reported in this publication was funded by the Italian Ministry of Health, RC-2025-2797263. Data availability at 10.5281/zenodo.15126372. No other potential conflict of interest relevant to this article was reported.

## Notes

KEY POINTS **QUESTION:** Do semiquantitative [^18^F]FDG PET parameters measured at 1 or 3 mo after CAR T infusion improve the prediction of clinical outcomes in patients with LBCL? **PERTINENT FINDINGS:** In a prospective single-center study of 61 patients, higher [^18^F]FDG PET parameters at both 1 mo and 3 mo after CAR T infusion were associated with inferior OS and PFS. In addition, these parameters at 3 mo showed a significant correlation with duration of response. **IMPLICATIONS FOR PATIENT CARE:** Incorporating semiquantitative PET measures into early postinfusion imaging may help clinicians identify high-risk patients sooner, potentially guiding more tailored treatment strategies and improving patient outcomes.
